# Impact of Molecular Testing in the Diagnosis of Thyroid Fine Needle Aspiration Cytology: Data from Mainland China

**DOI:** 10.1155/2014/912182

**Published:** 2014-01-29

**Authors:** Hui-qin Guo, Huan Zhao, Zhi-hui Zhang, Yan-li Zhu, Ting Xiao, Qin-jing Pan

**Affiliations:** ^1^Department of Pathology, Cancer Institute/Hospital, Chinese Academy of Medical Sciences and Peking Union Medical College, Beijing 100021, China; ^2^State Key Laboratory of Molecular Oncology, Department of Etiology and Carcinogenesis, Cancer Institute/Hospital, Chinese Academy of Medical Sciences and Peking Union Medical College, Beijing 100021, China

## Abstract

*Background*. The molecular work-up of thyroid nodules from fine needle aspiration samples has given clinicians a new level of diagnostic information. The aim of the present study was to evaluate the utility of molecular analysis in thyroid fine needle aspiration samples from a Chinese population. * Methods*. Specimens were collected from thyroid nodules by fine needle aspiration. Cytology diagnosis and genes analysis were performed and correlated with histology outcome. *Results*. A total of 83 patients with thyroid nodules were enrolled, including 20 benign lesions and 63 papillary carcinomas. BRAF and RAS mutations and RET/PTC gene rearrangements were found in 65.1%, 0%, and 1.6% of papillary carcinomas, respectively. No gene alterations were found in benign lesions. The combination of BRAF testing and cytology improved the accuracy of cytology from 69.9% to 89.2% (*P* < 0.05). Moreover, BRAF testing confirmed 82.4% of papillary carcinomas with suspicious cytology and identified 33.3% of papillary carcinomas with atypia cytology. *Conclusions*. Of the three candidate markers, BRAF testing showed diagnostic utility in fine needle aspiration. Combining BRAF testing with cytology improves the accuracy of fine needle biopsy. Those who have positive BRAF and malignant or suspicious malignant cytology can undergo thyroidectomy without a frozen section.

## 1. Introduction

Thyroid nodules are extremely common in the general population and are identified in 5% of patients by palpation and in 50% by ultrasound examination [1]. However, the majority of thyroid nodules are benign and best managed conservatively. Therefore, it is important to distinguish benign from malignant nodules. Fine needle aspiration (FNA) has emerged as the most successful diagnostic test in the management of thyroid nodules and has significantly decreased the number of thyroidectomies for benign thyroid lesions. However, FNA may also be nondiagnostic or may demonstrate indeterminate or suspicious cytologic features in 20–30% of all biopsies [2–5].

Of the various subtypes of thyroid malignancies, papillary thyroid carcinoma (PTC) is the most prevalent accounting for 80–85% of all thyroid malignancies. An accurate diagnosis of PTC is therefore important in determining the clinical management of thyroid nodules.

In recent years, our understanding of the molecular genetics of PTC has expanded significantly. Several studies of BRAF mutations alone or in combination with other oncogenes have increased the detection rate of PTC in thyroid FNA in Western countries [6–9]. However, there are few reports on molecular testing of FNA specimens in a Chinese population. The aim of this study was to determine BRAF, RAS (K-RAS, H-RAS, N-RAS), and RET/PTC (RET/PTC1, RET/PTC3) gene alterations in thyroid papillary carcinoma and evaluate the utilization of molecular testing in the diagnosis of FNA in a Chinese population.

## 2. Materials and Methods

### 2.1. Specimen Collection

This study enrolled patients with papillary thyroid carcinoma who underwent intraoperative frozen examination and had enough residual thyroid tissue for FNA at the Cancer Institute/Hospital, Chinese Academy of Medical Sciences (CAMS) between November 2010 and July 2011. Patients with benign thyroid nodules were also enrolled as negative controls. FNA was performed on surgically resected thyroid nodules by one of two cytopathologists (Dr. Guo and Dr. Zhao). Three passes were performed on each tumor. One pass aspirate was placed on a glass slide and immediately fixed in 95% alcohol for Hematoxylin and Eosin staining. The other two passes were performed for molecular testing. One pass was rinsed with CytoLyt solution (Cytyc, Marlborough, MA, USA) for DNA extraction and the other pass was rinsed with TRIZOL (Invitrogen, CA, USA) regent for RNA extraction. Cellular materials collected in CytoLyt and TRIZOL solution were frozen at −20°C. This study was reviewed and approved by the ethics committee of the Cancer Institute/Hospital, CAMS. All patients gave informed consent.

### 2.2. Cytological Diagnoses

The cytology slides were evaluated by two cytopathologists (Dr. Guo and Dr. Zhao) in a double-blinded manner. According to the TBS (The Bethesda System), cytological diagnoses were categorized as benign, AUS (atypia of undetermined significance), FN (follicular neoplasm), SM (suspicious malignant), and malignant.

### 2.3. DNA and RNA Extraction

Cells in CytoLyt solution were first centrifuged and then incubated in 500 *μ*L of DNA lysis solution [1 mg/mL proteinase K, 10 mmol/L Tris-HCl (pH 8.0), 0.1 mol/L EDTA (pH 8.0), and 0.5% (w/v) SDS] at 55°C for approximately 12 h; then the DNA was extracted by phenol and chloroform and stored at −20°C until use. RNA was extracted using TRIZOL reagent according to the user's manual and stored at −80°C until use. Both nucleic acids were quantified using a NanoDrop spectrophotometer. One microgram of total RNA was reverse transcribed using the SuperScript II Reverse Transcriptase kit (Invitrogen, CA, USA) following the manufacturer's instructions.

### 2.4. Polymerase Chain Reaction (PCR)

The primer sets for PCR amplification were designed as follows: BRAF (codon 600), 5′-TCATAATGCTTGCTCTGATAGGA-3′ (forward), 5′-GGCCAAAAATTTAATCAGTGGA-3′ (reverse). HRAS (codon 61), 5′-GTCCTCCTGCAGGATTCCTA-3′ (forward), 5′-ATGGCAAACACACACAGGAA-3′ (reverse). NRAS (codon 61), 5′-CCCCTTACCCTCCACACC-3′ (forward), 5′-TGGCAAATACACAGAGGAAGC-3′ (reverse), KRAS (codons 12/13), 5′-AAGGCCTGCTGAAAATGACTG-3′ (forward), 5′-GGTCCTGCACCAGTAATATGCA-3′ (reverse). GAPDH, 5′-GAAGGTGAAGGTCGGAGTC-3′ (forward), 5′-GAAGATGGTGATGGGATTTC-3′ (reverse). RET/PTC1, 5′-GGAGACCTACAAACTGAAGTGCAA-3′ (forward), 5′-CCCTTCTCCTAGAGTTTTTCCAAGA-3′ (reverse). RET/PTC3, 5′-CCAGTGGTTATCAAGCTCCTTACA-3′ (forward). PCR was prepared using TaKaRa Taq DNA polymerase (TaKaRa in a final volume of 50 *μ*L, with 1× TaKaRa Taq Buffer with Mg^2+^, 200 *μ*M of each dNTP, 200 nM of forward and reverse primers, 1.25 U of TaKaRa Taq, and 50–100 ng of DNA template). PCR cycling parameters were as follows: an initial denaturation at 95°C for 5 minutes, 35 cycles at 95°C for 30 seconds, 55–61°C for 45 seconds, 72°C for 30 seconds, and a final extension at 72°C for 10 minutes. Five microliters of PCR product was run on a 1.5% agarose gel to check the specificity of amplification, and 20 *μ*L was sent for direct sequencing with forward and reverse primers for each gene (SinoGenoMax Co., Ltd., China).

### 2.5. Statistical Analysis

Each patient's histological diagnosis was obtained from their case files. The results of histology were taken as the golden standard and cytological and molecular results were compared with histology. Statistical analysis of the data was performed with the chi-square test to compare the diagnostic ability of cytological and molecular testing. *P* < 0.05 indicated statistically significant results.

## 3. Results

A total of 83 cases with successful DNA and RNA extraction and PCR analysis were enrolled in our study, including 20 cases with benign lesions and 63 cases with PTC. Among the 63 cases with PTC, 54 cases were classic variant, 7 cases were follicular variant, and 2 cases were oncocytic variant. Of the benign lesions, 13 cases were goiter, 1 case was a follicular adenoma, 4 cases were granulomatous thyroiditis, and 2 cases were Hashimoto's thyroiditis.

The results of molecular testing are shown in Table 1. The most frequent gene mutation was BRAF. BRAF mutations were detected in 65.1% (41/63) of PTC lesions. However, BRAF mutations were not detected in the benign lesions (0/20).

**Figure 1:**
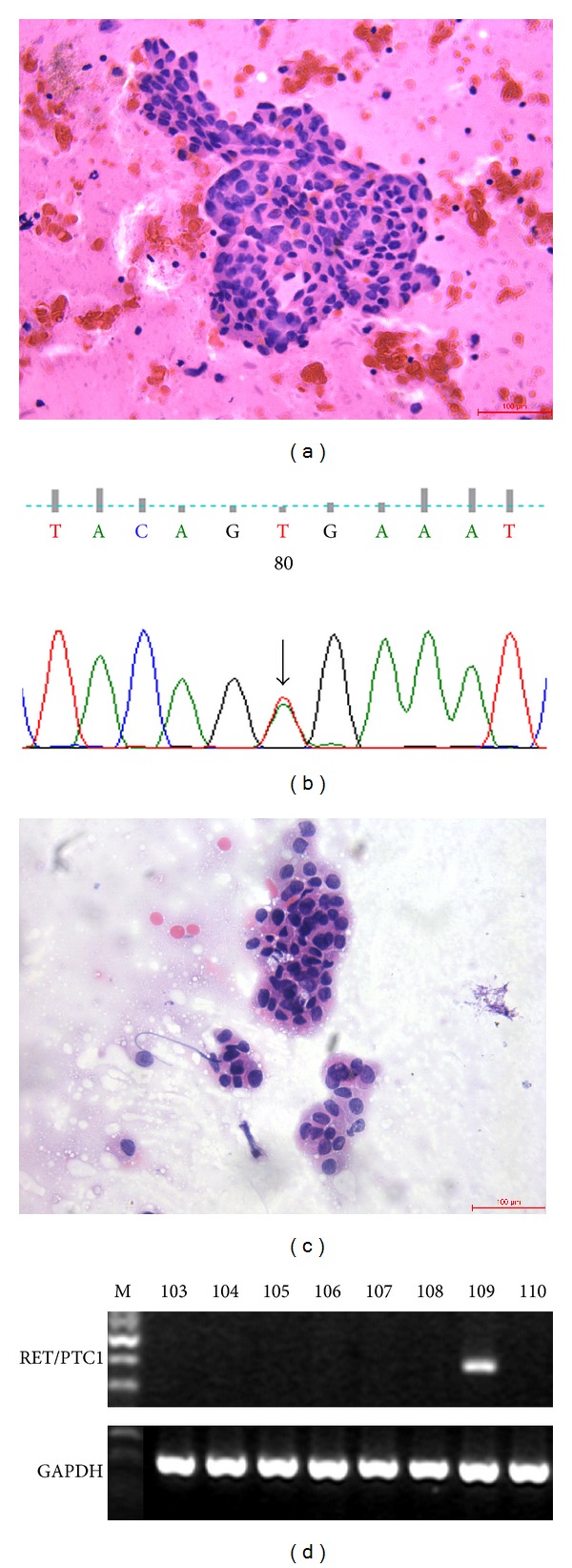
Correlation of cellular morphology and molecular results. (a) and (b) show a papillary carcinoma case with AUS cytological diagnosis: (a), Hematoxylin-eosin; (b), BRAF mutation. (Original magnifications ×200.) (c) and (d) show another papillary carcinoma case with AUS cytological diagnosis: (c), Hematoxylin-eosin; (d), RET/PTC1 rearrangement. (Original magnifications ×200.)

RET/PTC1 and RET/PTC3 in tumor cells were individually analyzed by RT-PCR. However, only RET/PTC1 rearrangement was found in 1 of 63 PTC cases (1.6%) (Figure 1). BRAF mutations were not found in patients with PTC and RET/PTC1 rearrangement. None of the benign lesions showed RET/PTC rearrangement. To screen for mutations in the RAS gene, N-RAS codon 61, H-RAS codon 61, and K-RAS codon 12 and 13 were analyzed. None of these three RAS mutations was observed in either malignant or benign lesions. The much lower prevalence of RET/PTC rearrangement and RAS mutation limited their use in FNA diagnosis.

Table 2 shows the cytology results of these 83 nodules and their corresponding histology. Eighteen cases were diagnosed as benign, 8 cases were diagnosed as AUS, 1 case was diagnosed as FN, 18 cases were diagnosed as SM, and 38 cases were diagnosed as malignant. All 38 malignant cases were confirmed by histology. Of the 18 SM cases, 17 were histologically malignant and 1 was goiter. One FN case was histologically goiter with adenomatous hyperplasia. Of the AUS cases, 6 cases were confirmed as PTC by histology, and the other two cases were goiter. In cytologically benign lesions, there were 2 PTCs, 1 adenoma, 9 goiters, and 6 cases of thyroiditis.

Using cytology alone, the diagnostic sensitivity, specificity, and accuracy for detecting PTC were 60.3% (38/63), 100% (20/20), and 69.9% (58/83), respectively. When we took suspicious malignant as the diagnostic threshold, the sensitivity, specificity, and accuracy for detecting PTC were 87.3%, 95.0%, and 89.2%. BRAF testing showed similar good specificity (100%) but better sensitivity (65.1%) and accuracy (73.5%) than cytology. When combining FNA cytology with BRAF testing, we obtained improved sensitivity (85.7% versus 60.3%, *P* < 0.05) and accuracy (89.2% versus 69.9%, *P* < 0.05) for malignancy. While suspicious malignant was considered a positive cytology outcome, combining BRAF testing increased the diagnostic accuracy from 89.2% to 91.6%. However, this improvement was not statistically significant (*P* > 0.05) (Table 3).

Table 4 shows BRAF mutations and cytological diagnoses in these 83 cases. In the group with suspicious cytology (*n* = 18), fourteen samples had BRAF mutations. At final histology, all BRAF mutated cases were PTC. BRAF testing confirmed 82.4% (14/17) of PTC with SM diagnosis. In the group with AUS cytology (*n* = 8), two samples had BRAF mutations, and both were confirmed as PTC by histology. BRAF testing detected 33.3% (2/6) of PTC in AUS cytology samples (Figure 1). In the group with benign cytology (*n* = 18), there were 2 cases of PTC and 16 cases of benign lesions. None had BRAF mutations.

## 4. Discussion

The main purpose of our study was to identify a useful panel of molecular markers in FNA specimens in mainland China as several studies have shown encouraging results using a molecular panel in FNA diagnosis in Western countries. In a study from USA, analysis of a panel of BRAF, RET/PTC, RAS, and PAX8/PPAR*γ* confirmed 60% (12/20) of cases with PTC from 513 follicular lesions of undetermined significance/atypia of undetermined significance cases [6]. Similar findings have also been reported in an Italian series in which cytology combined with a panel of BRAF, RET/PTC, and RAS analysis improved the total accuracy of FNA from 83.0% to 93.2% [7]. Therefore, we took BRAF, RAS (K-RAS, H-RAS, N-RAS), and RET/PTC (RET/PTC1, RET/PTC3) as candidate genes to evaluate the utility of the molecular panel in FNA specimens.

In our study, BRAF mutation was confined to PTC and was not detected in benign lesions. BRAF testing achieved an absolute specificity in PTC. The high specificity of BRAF testing is consistent with previous reports. A meta-analysis of 18 studies reporting the results of BRAF testing in 2766 FNA samples revealed 581 BRAF-positive samples. 580 cases were papillary carcinoma and only one case appeared to be benign. This nodule was pathologically diagnosed as “atypical nodular hyperplasia” [10]. The prevalence of BRAF mutations was 65.1% in PTC in our series which is similar to another large scale report from mainland China (62%) [11]. In a recent meta-analysis, the prevalence of BRAF mutations varied in different reports (28.8%–81.0%) and the average prevalence rate of BRAF mutations was 49.4% [12]. BRAF mutations in Chinese populations show a higher prevalence compared with the average prevalence rate of BRAF. Some studies indicate that iodine supplementation programs in China may have resulted in the observed high prevalence of BRAF mutations [11].

RET/PTC is another genetic alteration found in papillary carcinomas and was found in 1.6% of PTC in the present study. The reported RET/PTC prevalence in thyroid tumors varies greatly in different series and ranges from 0% to 73% in sporadic PTC [13–16]. The differences in reported RET/PTC prevalence may be attributed to ethnic and geographic variations and the different sensitivities of detection methods. Conventional RT-PCR is considered to be less sensitive but avoids the detection of nonclonal rearrangements [17]. Conventional RT-PCR was employed in the present study. The reported prevalence of RET/PTC rearrangements detected in Asia by conventional RT-PCR was low: Japan (0% to 9%) [18, 19], Korea (0% to 13%) [13, 20], and Taiwan (7%) [21]. The much lower prevalence of RET/PTC rearrangements in our study was consistent with these reports from Asia. In our series, BRAF mutations were not found in cases of PTC with RET/PTC1 rearrangement. This finding also supports the idea of mutual exclusivity between BRAF mutations and RET/PTC rearrangements in thyroid cancer [22].

Point mutations of the RAS genes are not restricted to a particular type of thyroid tumor and are observed in papillary carcinomas, follicular carcinomas, and follicular adenomas. Importantly, RAS mutations were identified in tumors which are difficult to diagnose by cytology alone, that is, the follicular variant of papillary carcinoma and follicular carcinoma. RAS mutation usually occurs in 10–20% of papillary carcinomas and 40–50% of follicular carcinomas. Moreover, it is also found in 20–40% of follicular adenomas [23]. To our surprise, we failed to identify any RAS mutations in thyroid lesions. This may be due to the small proportion of follicular tumors in this study. However, RAS mutation was not detected in the 63 cases of PTC, including 7 cases of follicular variant. A complete absence of RAS mutations was observed in papillary carcinoma in many published studies and most of these studies also originated from Asia, for example, Fukushima et al. (Japan) [24], Park et al. (Korea) [13], and Liu et al. (Taiwan) [25]. Thus, ethnic and geographic variations may greatly influence the prevalence of RAS gene alterations in thyroid tumors.

Our results show a higher prevalence of BRAF mutations and a much lower overall prevalence of RET/PTC and RAS alterations in thyroid tumors as compared to Western reports [12, 22, 23]. The much lower frequency of RET/PTC rearrangement and RAS mutations limited their utility in FNA diagnosis.

In our study, BRAF testing showed similar good specificity but better sensitivity and accuracy than cytology. However, we do not deem that it is reasonable to replace cytology examination with BRAF testing. In the study by Xing, BRAF testing was found to be robust and inexpensive and eliminated the need for cytology examination in nearly half of the patients with PTC undergoing FNA evaluation, which could result in substantial cost savings [22]. The majority (60%–80%) of FNA specimens in clinical practice are benign lesions and negative for BRAF mutations [26, 27]. This means that if BRAF testing is used for first screening of FNA specimens, about 80% of FNA specimens need an additional cytology examination. Therefore, we suggest performing BRAF testing as an additional method of cytology examination.

The combination of BRAF testing and cytology diagnosis improved the sensitivity and accuracy of detecting PTC compared with cytology alone. Molecular analysis was particularly advantageous in the indeterminate group, which included lesions with cellular atypia, follicular neoplasms, or suspicious malignant [28]. In the present study, BRAF testing detected 33.3% (2/6) of PTC in AUS cytology samples. Thus, for specimens with AUS cytological diagnosis, additional BRAF testing may improve the sensitivity of FNA examination. For specimens with SM diagnosis, BRAF testing confirmed 82.4% (14/17) of PTC with SM diagnosis. A definite malignant diagnosis of thyroid nodules may avoid intraoperative frozen examination. Frozen examination is the most common diagnostic method for suspicious thyroid nodules in China.

In conclusion, our findings confirm and extend previous results which demonstrated that BRAF testing increases the diagnostic accuracy of cytology, and patients with positive BRAF and malignant or suspicious malignant cytology can undergo thyroidectomy without a frozen section. However, the other two genes (RAS and RET/PTC) which have also been proved to be useful molecular tests in FNA specimens did not show significant utility. Larger studies, especially in patients with the follicular variant of PTC, are necessary to validate the current findings.

## Figures and Tables

**Table 1:** Correlation of molecular results and histological diagnoses.

Histological diagnoses	Molecular results					
	BRAF	RET/PTC1	RET/PTC3	K-RAS	N-RAS	H-RAS
Papillary carcinoma	65.1% (41/63)	1.6% (1/63)	0% (0/63)	0% (0/63)	0% (0/63)	0% (0/63)
Benign	0% (0/20)	0% (0/20)	0% (0/20)	0% (0/20)	0% (0/20)	0% (0/20)

**Table 2:** Comparison of cytological diagnoses and histological diagnoses.

Histological diagnoses (number of cases)	Cytological diagnoses				
	Benign	AUS	FN	SM	Malignant
Papillary carcinoma (63)	2	6	0	17	38
Adenoma (1)	1	0	0	0	0
Goiter (13)	9	2	1	1	0
Thyroiditis (6)	6	0	0	0	0

**Table 3:** Diagnostic values of FNA cytology, BRAF testing, or a combination of both for detecting PTC.

Diagnostic modality	SN	SP	PPV	NPV	AC
Cytology^a^	60.3%	100%	100%	44.4%	69.9%
Cytology^b^	87.3%	95.0%	98.2%	74.1%	89.2%
BRAF testing	65.1%	100%	100%	47.6%	73.5%
Combined^a^	85.7%	100%	100%	69.0%	89.2%
Combined^b^	90.5%	95.0%	98.3%	80.0%	91.6%

^a^Cytology diagnosis of malignant was taken as the diagnostic threshold.

^b^Cytology diagnosis of suspicious malignant was taken as the diagnostic threshold.

SN: sensitivity; SP: specificity; PPV: positive predictive value; NPV: negative predictive value; AC: accuracy.

**Table 4:** BRAF mutations and cytological diagnoses in 63 PTC and 20 benign lesions.

BRAF mutations	Cytological diagnoses of benign lesions					Cytological diagnoses of PTC				
	Benign	AUS	FN	SM	Malignant	Benign	AUS	FN	SM	Malignant
Positive	0	0	0	0	0	0	2	0	14	25
Negative	16	2	1	1	0	2	4	0	3	13
